# Relationship between job characteristics and music performance anxiety in collaborative pianists working in Slovenian music schools

**DOI:** 10.3389/fpsyg.2025.1582095

**Published:** 2025-06-05

**Authors:** Katarina Babnik, Žan Lep, Katarina Habe

**Affiliations:** ^1^Social Psychology and Policy Lab, Faculty of Arts, Department of Psychology, University of Ljubljana, Ljubljana, Slovenia; ^2^Educational Research Institute, Ljubljana, Slovenia; ^3^Department of Music Education, Academy of Music, University of Ljubljana, Ljubljana, Slovenia

**Keywords:** job demands, job resources, music performance anxiety, collaborative pianists, music education

## Abstract

**Introduction:**

Music performance anxiety (MPA) is a significant challenge for amateur and professional musicians, as well as music students. Systematic literature reviews highlight a lack of research on the role of contextual variables in MPA. This study contributes to existing knowledge by examining how job characteristics, particularly job demands and job resources, influence MPA among collaborative pianists.

**Method:**

A cross-sectional quantitative study was conducted on 94 collaborative pianists (24% of the population) working at all three levels of the Slovenian music education system. Data were collected through an online survey, including demographic characteristics, objective workload measures, and perceived job characteristics scales (cognitive job demands, emotional demands, job influence, role clarity, role conflict, and organizational justice), along with an adapted MPA scale. Confirmatory factor analysis and internal consistency analysis were conducted for each scale, followed by correlation analysis and a multiple linear regression model to predict MPA.

**Results:**

The study tested six hypotheses, with mixed support for the proposed relationships. The regression model explained 50% (*F* = 5.11; df1 = 10, df2 = 51, *p* < 0.001) of the variance in MPA, with perceived emotional demands (β = 0. 50; *t* = 3.57; *p* < 0.001) and role conflict (β = 0.38; *t* = 2.65; *p* = 0.011) playing significant roles in predicting MPA.

**Discussion and conclusions:**

The findings enhance the understanding of MPA in the job context and have practical implications for music school management. Future research should explore the interaction between job characteristics and individual psychological resources across diverse professional and cultural contexts with representative samples.

## Introduction

Many musicians are familiar with the anecdotal claim that a moderate level of performance anxiety can be beneficial for enhancing musical performance (Brugués, 2018). Extensive research has demonstrated, however, the potential detrimental effects of music performance anxiety (MPA) on both the physical and psychological wellbeing of musicians and music students (Kenny and Ackermann, 2015). Furthermore, contemporary research refutes the notion that MPA simply represents the opposite end of the spectrum of performance enhancement (Simoens et al., 2015).

As a psychological construct, MPA encompasses cognitive, emotional, and behavioral components (Kenny, 2011). It is most commonly examined through the framework of Barlow's (2000) theory of anxiety and anxiety disorders (Habe and Biasutti, 2024; Kenny, 2011; Orejudo et al., 2017). Systematic reviews of research conducted on professional and amateur musicians, as well as music students (e.g., Barros et al., 2022; Fernholz et al., 2019; Taborsky, 2007), provide evidence of various predictors, consequences, potential mechanisms, and possible treatments for MPA (Fernholz et al., 2019; Kenny, 2005). Most studies primarily focus on intra-individual factors associated with MPA, including self-evaluations (e.g., self-esteem, self-efficacy) and personality traits (e.g., trait anxiety, neuroticism, perfectionism; Barros et al., 2022; Habe and Biasutti, 2024). Although theoretical models of anxiety and MPA recognize the influence of environmental factors (Habe and Biasutti, 2024), research addressing the interaction between environmental factors (i.e., contextual influences) and individual characteristics remains limited (Barros et al., 2022).

From a performance context perspective, situational variables commonly examined include performance settings such as concerts vs. rehearsals (Barros et al., 2022; Robson and Kenny, 2017; Taborsky, 2007); jury vs. non-jury performances (Taborsky, 2007); solo vs. group performances (Barros et al., 2022); instrument-specific differences (Fernholz et al., 2019; Iusca and Dafinoiu, 2012); the timing of MPA across different phases of performance (before, during, and after; Taborsky, 2007); and the institutional culture of music education settings (Barros et al., 2022).

The present study aims to extend the literature by assessing the role of job context characteristics in MPA among a specific group of professional musicians—collaborative pianists. This research seeks to contribute to the existing body of knowledge on MPA in two key ways: (i) by examining the influence of job context, specifically job characteristics, on MPA, and (ii) by focusing on collaborative pianists employed in music education. The study first explores the unique job characteristics of collaborative pianists in Slovenian music education institutions, followed by a review of theories and studies on job characteristics and their impact on employees' wellbeing.

## The context of music education in Slovenia

Slovenia has a long tradition of music education spanning over two centuries. Within the European Union and globally, it is recognized as an example of best practice, offering a comprehensive public education system that spans elementary, secondary, and higher education (including undergraduate and postgraduate studies), and is funded by local communities and the state (Rotar Pance, 2019).

Like the general education system in Slovenia, the primary providers of music education are teachers at various levels, responsible for instructing students in instrumental performance, dance, singing, music theory, and composition. One unique aspect of music education, from the perspective of work organization and job classification, is the role of collaborative pianists. Across all levels of music education, accompaniment to solo instrumentalists, ensembles, singers, choirs, and dancers is provided by academically trained pianists.

According to national regulations (Pravilnik o normativih in standardih za izvajanje programa glasbene šole [Rulebook on Norms and Standards for the Implementation of the Music School Program], 2008), collaborative pianists must meet the same qualifications as other music teachers at different educational levels. In Slovenian music schools, accompaniment is typically performed by piano teachers, who, in addition to their teaching duties, also provide accompaniment as part of their workload. Alternatively, some academic pianists are employed exclusively as collaborative pianists, without teaching responsibilities.

## Collaborative pianists' job characteristics

A review of existing literature on the work of collaborative pianists reveals a gap in research on the relationship between job characteristics and MPA. Previous studies have primarily focused on describing the musical-functional and socio-emotional aspects of accompaniment (Roussou, 2017), the relationship between collaborative pianists and soloists (King and Roussou, 2017), the technical and social skills required for the role (Jordan Salingun, 2014; Mravunac Fabijanić, 2021), and the influence of negative expectancies and affective traits on MPA at different stages of performance (Gunders, 2023). While these studies highlight the specific nature of accompaniment, they do not provide a sufficient foundation for analyzing the role of job characteristics in MPA. Therefore, this study is grounded in the theoretical framework of job characteristics and their relationship to work-related wellbeing (Bakker and Demerouti, 2014, 2017; De Jonge and Dormann, 2017; Ji et al., 2024).

Every profession is defined by specific job characteristics that can be perceived as either (i) job demands or (ii) job resources (Bakker and Demerouti, 2014, 2017). Job demands refer to the physical, psychological, social, and organizational aspects of work that place strain on employees and may negatively impact their wellbeing. Conversely, job resources, which may not necessarily counteract job demands, include physical, psychological, social, and organizational aspects that facilitate task performance, mitigate burdens, and promote individual development (Bakker and Demerouti, 2014). These two factors influence employees through distinct yet interconnected pathways: job resources contribute to positive outcomes such as job satisfaction, engagement, motivation, and performance, while job demands contribute to negative outcomes related to job strain and health issues (Schaufeli and Taris, 2014).

Since the early research on job characteristics and employee wellbeing, key job characteristics that significantly impact wellbeing and health have been identified (Bakker and Demerouti, 2017; Frese and Zapf, 1994; Hackman and Oldham, 1976; Karasek, 1979; Luchman and González-Morales, 2013; Wilson et al., 2004). These include job variety, job complexity, autonomy and control, feedback, task and role clarity vs. role conflict, social support, and access to resources and information. While these job characteristics can serve as motivating factors, excessive job demands—if they exceed an individual's capacity to cope—can become sources of work-related stress, leading to anxiety and other negative consequences (Bakker and Demerouti, 2007). Depending on the nature of the work, job strain or excessive demands may manifest in cognitive (e.g., information processing), emotional (e.g., emotion regulation), or physical domains (De Jonge and Dormann, 2017; Ji et al., 2024).

The study of psychosocial job characteristics of musicians has a long tradition (Détári et al., 2020). Previous studies (Aalberg et al., 2019; Détári et al., 2020; Holst et al., 2012; Pihl-Thingvad et al., 2022; Vaag et al., 2014) report high job demands of (regularly employed and freelance) musicians and confirm the role of psychosocial work environment in their mental health. Compared to the general workforce population, professional orchestra musicians report higher emotional demands, lower job influence and social support, and reduced job satisfaction (Holst et al., 2012). A large-scale epidemiological study comparing musicians and general workforce reports higher perceptions of job control, but also higher job demands, lower social support, lower effort-reward balance, greater work-family conflict, and reduced motivation in musicians (Détári et al., 2020). Symptoms of psychological distress, including anxiety and depression, are prevalent among musicians and are influenced by both job demands and personal characteristics (e.g., neuroticism, low mastery) (Aalberg et al., 2019).

## This study

The objective of this study was to assess the job characteristics of collaborative pianists employed in Slovenian music schools and to determine their role in music performance anxiety (MPA). Specifically, our goals were: (i) to identify job characteristics that function as job demands (psychosocial and organizational stressors) and job resources (psychosocial and organizational factors that support employees, enhance performance, and mitigate the negative effects of job demands) and (ii) to examine the role of these job characteristics in the MPA experienced by collaborative pianists. The study was guided by the following research question: How do job characteristics, categorized as job demands and job resources, relate to MPA?

Besides addressing a gap in the literature, this study was also guided by the promise of practical implications. The Društvo klavirskih pedagogov Slovenije - EPTA (n.d.) initiated and supported the study due to the challenges observed in the job characteristics of collaborative pianists working in Slovenian music education institutions. These challenges include workload issues stemming from the dynamic nature of their work (high flexibility demands), unevenly distributed working hours, increasing expectations for performance quality (higher difficulty levels of repertoire across all education levels), and the necessity for additional engagement beyond regular working hours (practice and independent preparation). Consequently, the study aimed to address practical concerns within the field of music education.

**Figure 1:**
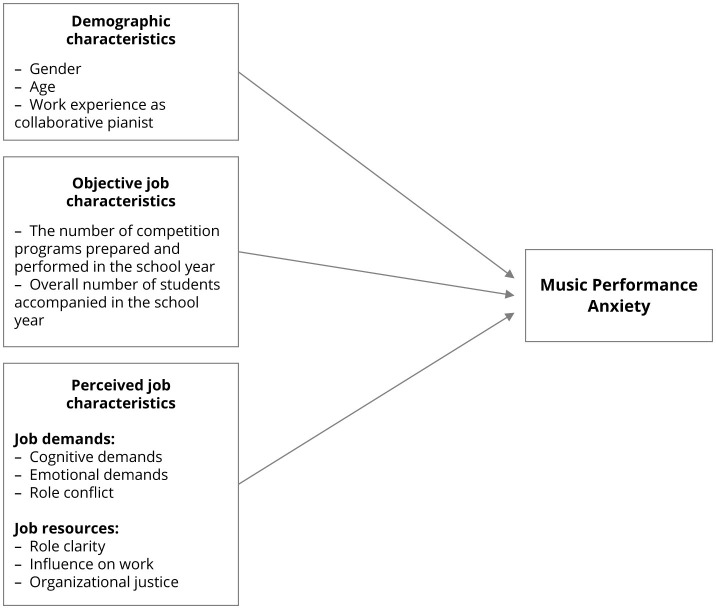
Set of MPA predictors included in the study.

Figure 1 presents constructs analyzed in this study. The empirical model was developed based on findings from prior research and theoretical frameworks (e.g., Bakker and Demerouti, 2014, 2017; Schaufeli and Taris, 2014; Van den Tooren and de Jonge, 2011) as well as systematic reviews of MPA studies (Barros et al., 2022; Fernholz et al., 2019; Habe and Biasutti, 2024; Taborsky, 2007).

The first set of variables included in the empirical model pertains to individual demographic characteristics: gender, age, and work experience. Although findings are inconsistent (e.g., Iusca and Dafinoiu, 2012; Kenny et al., 2004), previous studies suggest that female musicians report higher MPA levels (Butković et al., 2022; Habe and Biasutti, 2024; Yondem, 2007). Another demographic factor influencing MPA is age. Research indicates that MPA scores increase with age in young musicians (Dempsey and Comeau, 2019; Patson and Osborne, 2016). However, this relationship is not linear, with MPA levels rising during adolescence (LeBlanc, 2021) and middle adulthood (Fernholz et al., 2019). Increased experience with musical performance and preparation among those who continue their careers post-formal education may significantly impact MPA levels, as indicated in previous studies (Taborsky, 2007). Accordingly, our hypotheses regarding demographic characteristics and MPA among collaborative pianists are as follows: Female collaborative pianists experience higher MPA levels (H1), while age (H2) and years of work experience are negatively correlated with MPA (H3).

The second and third sets of variables pertain to job characteristics. Two types of job characteristics were defined based on data collection methods: (i) objective workload data for collaborative pianists in the current academic year and (ii) subjective (perceived) job characteristics.

The objective workload characteristic included in the analysis measured the number of students collaborative pianists accompanied in national and international competitions, where students performed in front of a jury. Previous studies confirm that competitive or jury-based performance situations are particularly stressful for musicians (Habe and Biasutti, 2024; Taborsky, 2007). Consequently, the total number of competition programs prepared and performed in the school year represents a job demand. We hypothesized that an increased workload associated with jury-based performance contexts and overall job demands, operationalized as the number of students accompanied in a school year, contributes to higher MPA levels (H4).

Perceptions of job-level and institutional-level characteristics serve as evaluative mediators between objective environmental conditions and individual responses (James et al., 2008). Perceived job characteristics constitute “psychologically meaningful representations” (Parker et al., 2003, p. 390) of the work environment, shaping attitudes, behaviors, and long-term wellbeing. The relationships between objective environmental characteristics, perceived job characteristics, and individual responses indicate that both objective and perceived job characteristics are valid predictors of employees' cognitive and affective responses (Algera, 1983). However, perceptions are not direct reflections of objective job conditions; they are influenced by individual traits and life experiences (Mandler, 1982). This explains the variability in convergence between different sources of job-related information (e.g., employee perceptions, observer perceptions, and objective data; Spector and Jex, 1991).

In line with the results of previous studies identifying specific job demands and resources among musicians compared to the general workforce, such as cognitive and emotional demands, effort-reward balance (justice), and job autonomy (Détári et al., 2020; Holst et al., 2012), as well as the specific demands of collaborative pianists highlighted by the Slovene Piano Teachers Association (e.g., role conflict vs. role clarity), the perceived job characteristics assessed in this study included cognitive and emotional demands, job autonomy (influence over work), role conflict, and role clarity. Emotional demands are associated with increased distress and reduced job satisfaction (Pugliesi, 1999). Quantitative job demands (workload), emotional demands, and time constraints predict work-related exhaustion (Tuxford and Bradley, 2015), while high job demands (workload and emotional burden) contribute to distress, burnout, and increased anxiety levels (Santa Maria et al., 2018). Prior research confirms that job demands can exacerbate anxiety through pathways involving distress and burnout. Perceived organizational justice was also included as a potential job resource for collaborative pianists. This construct reflects employees' perceptions of fairness and recognition within an organization, which is fundamental to social structures (Kivimäki et al., 2003, p. 27). Prospective studies (e.g., Kivimäki et al., 2003) confirm that perceptions of procedural and interpersonal justice independently impact employee health. Additionally, a systematic review of prospective studies (Ndjaboué et al., 2012) highlights the role of organizational justice in employees' mental and physical wellbeing. Organizational justice serves as an institutional job resource that mitigates the negative effects of job demands by fostering perceptions of social support (Huang et al., 2019).

Based on the findings above, we hypothesized that perceived cognitive and emotional demands, as well as perceived role conflict, positively contribute to MPA among collaborative pianists (H5), thereby acting as job demands. Conversely, perceived role clarity, job autonomy, and organizational justice were hypothesized to negatively correlate with MPA, functioning as job resources (H6).

## Methods

The study was conducted as a cross-sectional quantitative investigation involving formally employed pianists who serve as collaborative pianists at the primary, secondary, and tertiary levels of the Slovenian music education system. Prior to conducting the research, approval was obtained from the Ethics Committee of the Faculty of Arts, University of Ljubljana (reference number 328-2023). The online survey was designed based on validated instruments from previous international studies. Before data processing, psychometric validation (including reliability and construct validity) of the instruments was performed.

The following sections provide a detailed description of the participant sample, the recruitment process, sample characteristics, the measurement instruments, and the data analysis procedure.

### Sample

The sample comprised pianists employed at all three levels of music education in Slovenia who were actively employed (regardless of the percentage of employment) during the study period (June–August 2023) at educational institutions listed in the school directory as providers of publicly recognized music education curricula. Recruitment was conducted through the formal channel of the Slovenian Music School Association (SMSA), which represents music education institutions in Slovenia. SMSA disseminated the online survey to the principals of primary and secondary music schools and to the deans of higher education institutions that employ collaborative pianists, requesting them to invite their colleagues to participate. Additionally, the EPTA Slovenia informed its members about the study and encouraged their participation. Based on EPTA Slovenia's data, it was estimated that approximately 400 pianists worked as collaborative pianists in music education institutions in 2023 (study population). The expected sample size was thus estimated at 150 participants.

A total of 365 pianists working as collaborative pianists in music education accessed the online survey. Only 94 participants, however, representing approximately 24% of the population of collaborative pianists working in the formal music education system, completed at least 50% of the survey items, resulting in the final realized sample.

The final sample comprised 94 participants, all of whom either worked exclusively as collaborative pianists or combined piano teaching with an accompanying role. The sample was composed of 75% women, with an average age of 41.5 years (SD = 10 years; min = 25, max = 61) and an average work experience in job position of 17.2 years (SD = 10.7 years; min = 1, max = 38).

Most participants were employed on full-time, permanent contracts (87.5%), while 75.8% had additional weekly teaching responsibilities. The majority were employed at the primary music education level (62.4%), followed by those with combined employment at both the primary and secondary levels (20.4%) and those working in higher education (9.5%). The least represented group comprised participants employed exclusively at the secondary education level (3.2%).

### Research instruments

The online survey included multiple instruments assessing job perceptions, objective job characteristics, demographic data, MPA, and perceptions regarding possible changes in the organization and role of accompaniment within the Slovenian music education system. For the objective of this study, the following instruments were used:

#### Objective job characteristics

Participants provided information on their employment (primary, secondary, tertiary education; employment type) and any additional work outside their regular employment (e.g., freelance contracts, occasional jobs, or amateur performances). They also reported the number of programs they have prepared and performed as collaborative pianists at national and international competitions during the 2022/2023 school year. These competitions typically involved performances before a jury composed of national and international music professionals. Participants specified the number of competition programs they prepared and performed, as well as the total number of students they accompanied throughout the school year.

#### Perceived job characteristics

Job perceptions were assessed using selected subscales from the *Psychosocial Aspects of Work - The Third Version of the Copenhagen Psychosocial Questionnaire (COPSOQ-III)*, developed by Llorens-Serrano et al. (2020). Since 2007, this instrument has been updated through the international COPSOQ network. The following segments were used in this study:

– Cognitive demands (3 items from the original 4-item scale)
– Emotional demands, including the requirement to suppress emotions (4 items from the original 7-item scale)
– Influence at work (6 items)
– Role clarity (3 items)
– Role conflict (2 items)
– Organizational justice (4 items)

Participants rated each item on a five-point Likert type scale, with response options ranging from 1 (never/almost never) to 5 (always) or 1 (to a very small extent) to 5 (to a very large extent) depending on the specific construct.

Following an evaluation of the data distribution for each scale, confirmatory factor analysis (CFA) was conducted using the Maximum Likelihood Method to assess the dimensional structure and internal consistency of the selected perceived job characteristics. Two separate CFAs were performed. The first examined the dimensional structure of scales measuring job-level characteristics (cognitive demands, emotional demands, influence at work, role clarity, and role conflict). The second assessed the dimensional structure of organizational-level characteristics, specifically perceived organizational justice.

CFA confirmed 5-dimensional structure of perceived job-level characteristics scale (χ^2^ = 134, *df* = 109, *p* = 0.054; CFI = 0.954; TLI = 0.943; SRMR = 0.074; RMSEA = 0.052, 90% CI [0.00, 0.08]), with χ^2^ exceeding *p* = 0.05, and satisfactory CFI, TLI, and SRMR values, following Byrne's (1994) recommended criteria. The RMSEA slightly exceeded the 0.05 threshold, which is expected given the small sample size. The subscales show appropriate internal consistency: cognitive demands (α = 0.62), emotional demands (α = 0.68), influence at work (α = 0.84), role clarity (α = 0.88), and role conflict (α = 0.78). Similarly, the organizational justice scale exhibited adequate fit measures, except for the RMSEA, and confirmed one dimensional structure of the organizational justice scale (χ2 = 4.42, *df* = 2, *p* = 0.110; CFI = 0.989; TLI = 0.966; SRMR = 0.019; RMSEA = 0.126, 90% CI [0.00, 0.08]) with high internal consistency (α = 0.91).

*The level of music performance anxiety (MPA*) was assessed using selected items from the Kenny Music Performance Anxiety Inventory (K-MPAI; Kenny, 2011, 2023). Specifically, four items from the Proximal Somatic Anxiety and Worry About Performance subscale of the K-MPAI (Kenny, 2023) were utilized to measure participants' self-perceptions of anxiety related to their performance as collaborative pianists. Participants rated each item on a five-point Likert type scale: 1 (not at all characteristic of me), 2 (partially uncharacteristic), 3 (neither characteristic nor uncharacteristic), 4 (partially characteristic), and 5 (very characteristic). Notably, for this study, the response scale was modified from the original scale defined in the K-MPAI (Kenny, 2023). The four items were selected based on their relevance to identification of the level of anxiety and worry before and during collaborative performance: *Prior to, or during a performance, I get feelings akin to panic; I never know before a concert whether I will perform well; During a performance I find myself thinking about whether I'll even get through it; I am often concerned about a negative reaction from the audience*. The response scale was adjusted to ensure consistency with other instruments used in the survey and to enhance clarity for participants. The CFA (Maximum Likelihood Method) confirms one-dimensional structure of the selected four items, with χ^2^ = 3.73, *df* = 2, *p* = 0.155; CFI = 0.991; TLI = 0.973; SRMR = 0.017; RMSEA = 0.106, 90% CI [0.00, 0.272]). Alfa coefficient of internal reliability for the four-item scale was 0.90.

### Procedure

Data collection was conducted online via the 1KA platform (https://1ka.arnes.si/). Participants were informed about all key aspects of the study. The introductory page provided detailed information regarding the study, including the contact details of the study coordinator. Participants provided informed consent before proceeding with the survey. The collected data were systematically organized and analyzed using Jamovi (The Jamovi Project, 2023). The missing data were excluded pairwise and were not imputed. First, descriptive analyses, including data distribution analysis, were conducted. Confirmatory factor analysis (CFA) and internal consistency analysis were then performed for each of the scales used. Third, Pearson correlation coefficients were calculated, followed by testing a multiple linear regression model (with assumptions checking) to examine the relationships among variables and their role in predicting MPA.

## Results

Table 1 presents the descriptive statistics for the scales used in this study. Objective job characteristics of collaborative pianists were assessed through open-ended questions. On average, participants accompanied 34 students (M = 33.8, SD = 21.38) and prepared and performed seven competition programs (M = 6.7, SD = 7.35) during the 2022/2023 school year.

**Table 1:** Descriptive statistics for objective and perceived job characteristics included in the study.

**Scales**	**N**	**M**	**SD**
Cognitive demands	83	3.69	1.044
Emotional demands	77	3.85	0.773
Influence at job	80	2.66	0.886
Role clarity	76	3.98	0.922
Role conflict	75	2.86	1.105
Organizational justice	73	3.02	1.150
Music performance anxiety	77	3.32	1.278

N, number of respondents; M, average; SD, standard deviation.

Among all assessed job characteristics, participants reported the lowest levels for perceived job influence (*M* = 2.66, SD = 0.886). This includes perceptions of limited autonomy in decision-making and job planning, specifically regarding when, how, and with whom they work. Conversely, participants reported high emotional demands associated with their role (*M* = 3.85, SD = 0.773). They primarily perceived their work as collaborative pianists to be emotionally demanding, requiring them to suppress their opinions and emotions while maintaining a courteous and respectful attitude toward all colleagues and collaborators.

On the other hand, participants reported relatively high levels of role clarity (*M* = 3.98, SD = 0.922). They expressed a clear understanding of job expectations and performance requirements, as well as well-defined goals associated with their role.

Table 2 shows correlations between MPA and studied demographic, objective, and perceived (subjective) job characteristics; cognitive demands, emotional demands, role clarity and conflict, and organizational justice are all correlated with MPA. Cognitive and emotional demands and role conflict represent job demands and are positively connected with the level of MPA. Hypotheses 2 and 3 stating that demographic variables (age and working experience in job position) and objective job characteristics correlate with the level of MPA are not confirmed since these variables were not correlated. The difference in the level of MPA between males and females was tested with Student's *t*-test. The results show that female (*N* = 57; *M* = 4.00; SD = 1.24) perceive higher levels of MPA than males (*N* = 17; *M* = 2.75; SD = 2.25; *t* = 2.15; *df* = 72; p = 0.035), supporting our H1.

**Table 2:** Pearson correlation coefficient between MPA and studied variables.

**Variables**	**Pearson correlation with MPA**
Age	−0.132
Working experience in job position	−0.023
Number of competition programs prepared and performed in 2022/2023	0.154
Number of students accompanied throughout the 2022/2023	0.094
Cognitive demands	0.267^*^
Emotional demands	0.631^**^
Influence at job	−0.158
Role clarity	−0.239^*^
Role conflict	0.526^**^
Organizational justice	−0.329^**^

* *p* ≤ 0.05.

** *p* ≤ 0.001.

*N* = 80.

The leading research question of this study was how do job characteristics that represent job demands and job resources relate to MPA? As defined in Figure 1, our regression model also included demographic characteristics of the participants. Although correlation coefficients show pairwise relationships between variables, multiple linear regression identifies how the variables interrelate in their prediction of dependent variables. As in everyday job situations, job and individual characteristics are not influencing individuals as separate factors, but rather codetermine individuals' reactions. Since the variables age and years of work experience in job position were highly correlated (*r* = 0.962; *p* < 0.001), only years of work experience in job position was included in the regression model. Table 3 shows the coefficients obtained in testing a multiple linear regression model. The model explained 50% of the variance in MPA scores (*R*^2^ = 0.500) and was stable (*F* = 5.11; *df1* = 10, *df2* = 51, *p* < 0.001). As shown in Table 3, several studied variables that show correlation with MPA (Table 3) do not contribute individually to MPA when studied in a complex multiple linear model, and vice versa. Of the demographic variables, gender (ß = 0.109; *t* = 0.79; *p* = 0.004) and work experience (ß = 0.18; *t* = 1.47; *p* = 0.147) were not predictive of MPA. Objective job characteristics (number of students accompanied in the school year, number of competition programs in the school year) had no predictive power. From the group of perceived job characteristics, only emotional demands (ß = 0. 50; *t* = 3.57; *p* < 0.001) and role conflict (ß = 0.38; *t* = 2.65; *p* = 0.011) predicted MPA, with emotional demands contributing the highest proportion of variance in MPA.

**Table 3:** MPA prediction model estimates.

**Predictor**	**ß**	***t***	***p***
Gender	0.09	0.79	0.436
Work experience in job position	0.18	1.47	0.147
Number of students accompanied in the school year	0.08	0.64	0.524
Number of competition programs in the school year	0.13	1.08	0.284
Cognitive demands	−0.13	−1.07	0.290
Emotional demands	0.50	3.57	< 0.001
Influence at job	0.21	1.65	0.105
Role clarity	0.08	0.58	0.562
Role conflict	0.38	2.65	0.011
Organizational justice	−0.12	−0.89	0.377

ß, standardized beta; *t, t*-test statistics; *p*, probability value.

## Discussion and conclusions

Previous studies have primarily focused on the role of individual-level characteristics (Barros et al., 2022; Habe and Biasutti, 2024) and the specific characteristics of performance contexts in relation to MPA (Barros et al., 2022; Robson and Kenny, 2017; Taborsky, 2007). This study contributes to the understanding of MPA by identifying the role that job characteristics of professional musicians working as collaborative pianists in music schools play in their perceived levels of MPA.

In alignment with the study's objectives, our research is grounded in work and organizational psychology theories, which explain the influence of job characteristics on employee wellbeing and the mechanisms underlying this relationship (Bakker and Demerouti, 2017; Frese and Zapf, 1994; Hackman and Oldham, 1976; Karasek, 1979; Luchman and González-Morales, 2013; Wilson et al., 2004). The study was conducted with approximately 24% of collaborative pianists employed in Slovenian primary and secondary music schools, as well as higher education institutions. While the raw sample size is small considering the established standards in psychological research, we believe it is still meaningful in terms of the population share we were able to survey.

The empirical model developed in this study tested six hypotheses, though not all were supported by the results. We hypothesized that female collaborative pianists experience higher levels of MPA (H1), while age (H2) and years of work experience (H3) are negatively correlated with MPA. When examining these three variables independently, only gender differences in MPA were confirmed. In the multiple linear regression model, however, gender did not have a significant explanatory contribution. Previous studies confirm that both age and years of experience play a role in MPA. MPA tends to peak during adolescence (Taborsky, 2007), whereas increased experience leads to mastery and subsequently lower MPA levels (Taborsky, 2007). Given that our sample consists predominantly of young adults and mid-career collaborative pianists (working age population), absence of correlations between age and MPA and between work experience and MPA may be attributable to this demographic composition.

The second set of hypotheses relates to job characteristics. Increased workload, operationalized as the number of jury performance contexts and the overall number of students accompanied, does not correlate with MPA and does not contribute significantly to the MPA prediction model. Although workload has been a key focus in job-related stress research from early studies (Frese and Zapf, 1994; Karasek, 1979) to recent investigations (Santa Maria et al., 2018; Tuxford and Bradley, 2015), our findings do not support the role of quantitative demands in MPA. Therefore, the hypothesis 4 was not supported in our study.

The third set of hypotheses examines perceived job characteristics. We hypothesized that cognitive and emotional demands, as well as perceived role conflict, would positively contribute to MPA (H5), while perceived role clarity, job influence, and organizational justice would negatively correlate with MPA (H6). The correlations among these variables support our hypotheses. Role clarity and organizational justice show negative correlations with MPA, serving as job resources that help employees manage work-related demands (Bakker and Demerouti, 2017). Conversely, cognitive and emotional demands, along with role conflict, correlate positively with MPA, functioning as job demands that may negatively impact productivity and wellbeing by increasing anxiety levels (Santa Maria et al., 2018).

However, the regression model indicates that only emotional demands and role conflict significantly contribute to MPA. In multiple linear regression models, where variables are analyzed collectively, their effects are assessed in relation to one another. Our findings suggest that, when considered alongside other studied variables, emotional demands and role conflict remain strong predictors of MPA. These results align with research on the unique characteristics of collaborative work and the emotional demands associated with such roles (Jordan Salingun, 2014; King and Roussou, 2017; Roussou, 2017). Also in the teaching profession, emotional demands are frequently studied and confirmed as predictors of job-related stress (Tuxford and Bradley, 2015). Collaborative pianists assume multiple roles (Jordan Salingun, 2014; King and Roussou, 2017; Mravunac Fabijanić, 2021; Roussou, 2017), serving as mentors and co-performers. In music schools, where they work with students of varying ages and performance competencies, role conflict may arise in relation to primary instrument teachers. Ambiguities in planning, organizing, and executing rehearsals and performances likely contribute to increased stress levels. Especially if the roles and expectations of both collaborative pianists and primary instrument teachers are not clearly defined, pianists may face uncertainty about who is responsible for key aspects of the preparation and performance process. For instance, decisions related to musical interpretation, rehearsal scheduling, and the assessment of students' performance may fall into a gray area, with both the collaborative pianist and the primary teacher perceiving different degrees of authority and responsibility. This ambiguity can give rise to conflicting role expectations. The pianist may be expected to provide support while simultaneously performing artistic judgment but without the formal authority to make final decisions. Furthermore, pianists may receive inconsistent or even contradictory feedback from students and teachers, which can reduce their perceptions of control over the collaborative process.

An important question is also why job resources did not emerge as significant predictors of MPA. One possibility is that job resources, while important for overall well-being, may be less directly influential in acute performance-related anxiety, which tends to be more sensitive to demands (stressors) than to protective or buffer factors. This hypothesis, however, needs further investigation in future research.

To summarize the results and compare them with the hypotheses that guided the research, we find that when examining the independent roles of variables (i.e., correlations between the studied variables and the level of MPA), Hypothesis 1 (H1) can be confirmed. Levels of MPA differ by gender (with women reporting higher levels of MPA) and are positively associated with cognitive and emotional demands, as well as perceived role conflict (H5), and negatively associated with role clarity, job influence, and organizational justice (H6). A joint analysis of the variables (multiple regression analysis) revealed that only two variables (emotional demands and role conflict) significantly predict the level of MPA. Since these variables tend to co-occur in workplace settings, the results of the regression analysis are particularly relevant for our conclusions. These findings indicate that job demands, specifically emotional demands and role conflict (H6), play a significant role in predicting MPA levels among the studied sample of collaborative pianists.

The regression model, incorporating both demographic and job characteristics, explains half of the variance in MPA levels (50%). This finding underscores the importance of job characteristics in explaining MPA and highlights the need to include them in future research, particularly in conjunction with individual characteristics that may serve as psychological resources, such as self-efficacy and self-esteem (Xanthopoulou et al., 2007). Additionally, this study has practical implications for music school administration. Job-related stress and anxiety among collaborative pianists could be mitigated by reducing role conflict through enhanced collaboration between pianists and primary instrument teachers. Strategies such as joint planning of rehearsals and concerts, fostering a climate of mutual respect, and recognizing the complexity and significance of the collaborative pianist's role in music education may help alleviate job-related stress. Through clearer communication, mutual understanding, and a more balanced distribution of responsibility, expectations regarding rehearsal planning, interpretative decisions, and performance preparation can be clearly defined. This would help to prevent the confusion that often arises when roles are ambiguous or overlapping (role conflict).

While highlighting the strengths of this study and its implication, we must also honestly address some of its limitations; the primary being the small sample size, which restricts the applicability of complex analyses and multiple regression models involving numerous dependent variables. In addition, the sample consist mainly of participants employed on the level of primary music education and has limited representation of secondary and tertiary level participants. Moreover, the cross-sectional design of our study allows only conclusions about correlations and not causality between the studied variables. Consequently, the conclusions drawn should be interpreted with caution. Nonetheless, this study provides insights into future research directions on MPA, particularly in professional musicians working within structured institutional systems. Future studies should explore more complex interactions between job characteristics and individual traits in predicting MPA (such as moderating effects of psychological resources (e.g., self-efficacy) on the relationship between job demands and MPA), examining diverse samples of professional musicians across different cultural and national contexts to establish more valid and reliable conclusions on the interplay between contextual and individual factors in MPA.

## Data Availability

The raw data supporting the conclusions of this article will be made available by the authors, without undue reservation.
